# Hybrid Bacterial Colony Optimization and Particle Swarm Optimization for load balancing in fog computing

**DOI:** 10.1371/journal.pone.0347176

**Published:** 2026-05-08

**Authors:** Aryan Kumar, Punit Gupta, Rohit Verma

**Affiliations:** 1 School of Computing, National College of Ireland, Dublin, Ireland; 2 Pandit Deendayal Energy University, Gandhinagar, Gujrat, India; 3 Technological University Dublin, Dublin, Ireland; King Abdulaziz University, SAUDI ARABIA

## Abstract

Fog computing minimizes latency and bandwidth consumption by processing data near the source, but it has the challenge of workload balancing across the dynamic and resource limited fog nodes. Uneven task assignment can result in bottlenecks, idle resources, and lowered Quality of Service (QoS) standards. In this work, we introduce a hybrid metaheuristic load balancing algorithm for fog computing by combining the Bacterial Colony Optimization (BCO) and the Particle Swarm Optimization (PSO) techniques to enhance load balancing. BCO has good solution space exploration capabilities, whereas PSO has the advantage of quick convergence; the hybrid takes advantage from both to achieve reduced makespan with better VM usage. The proposed algorithm has been developed in the Python language, with original BCO and hybrid modules, along with a standard PSO executable. The program has been tested on a synthetic offline task–VM dataset generated by CloudSim 6.0 with size ranges from 100 to 10,000 tasks and poison distribution of variety of tasks arriving, with identical experiment settings. The results indicate the hybrid BCO–PSO to exhibit significant makespan reduction with increased VM utilization compared to the individual BCO, PSO, as well as the Adaptive Inertia Weight Particle Swarm Optimization (AIW–PSO) algorithms for most test scenarios, with faster convergence for high workload scenarios. In the high-load cases, the hybrid reduced makespan by 32.76% compared to AIW–PSO for 5000 tasks and by 35.79% compared to AIW–PSO for 10000 tasks. These experimental results indicate that the proposed hybrid algorithm can be an effective, adaptive solution for task allocation in fog-inspired computational scheduling scenarios. This evaluation focuses on computation-side task scheduling using a synthetic task–VM model and keeping network-level factors such as latency or bandwidth idle.

## 1. Introduction

**Fig 1:**
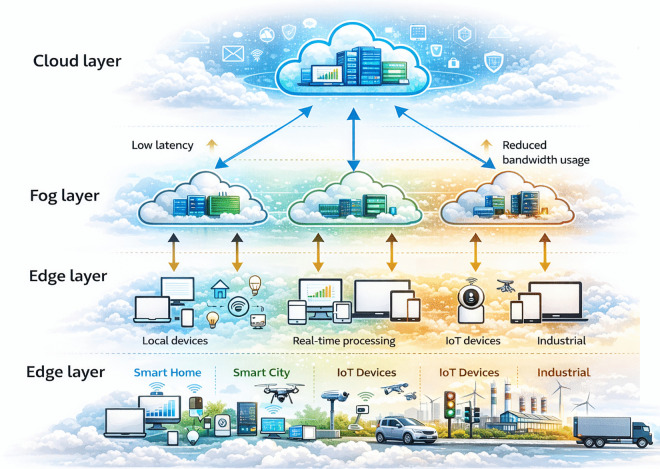
Fog computing architecture.

Fog computing is the decentralised approach that extends the capabilities of the cloud computing through bringing the computation task, storage of data, and network services closer to the source of the data [1]. Unlike the traditional centralised cloud approach this model processes data locally rather than transferring all information to the cloud. This helps to reduce the latency and lowers the bandwidth usage that is very critical factors for Internet of Things (IoT) applications [2]. A common fog model is a three-layer architecture consisting of IoT/end devices, a fog layer (fog nodes/gateways), and the cloud layer [1]. In this architecture, many interconnected devices are organised into fog nodes and this node performs computational tasks for the connected end devices. These fog nodes do the filtering, aggregate and analyse the data near its source and reduce the workload on cloud resources and improving real-time responsiveness, as illustrated in Fig 1.

Handling and analysing data at the fog layer certainly removes the need to send all raw data to distant cloud servers. As a result, fog computing performs efficiently in terms of latency, energy consumption, network traffic, and task distribution. However, the changing workloads and varying fog resources make effective load balancing a continuous challenge.

### 1.1. Research motivation and background

Although fog computing certainly decreases the latency for the applications of IoT in the real-time use cases, but it is still facing issues with the scheduling task and of load balancing. This still remains a limitation due to the changing workload in the fog environment and the limited resources available at the fog nodes [1].

As some traditional task scheduling methods like Round-Robin could not provide good performance with these changing workloads conditions. This caused an issue of long queues of tasks with high energy wastage and poor Quality of Service (QoS) [2]. Because of these, the researchers have chosen the metaheuristic algorithms as an alternative method that can be used to design and accommodate these limitations. Among these, Particle Swarm Optimization (PSO) algorithm is well known for its simplicity and fast convergence nature [3], but it often suffers from premature convergence problems and poor population diversity in later stages of the search space. Bacterial Colony Optimization (BCO), on the other hand gave a strong exploration nature through the mechanisms like chemotaxis and migration, which is better for more exploration, but this algorithm is likely to converge very slowly [4].

To address these disadvantages, some hybrid metaheuristic methods have been explored in different combinations and methods. As [5] showed on combining the BCO’s exploratory strength with PSO’s fast convergence property will improve the accuracy and stability in the tasks. Similarly, hybrid algorithms like PSO–Firefly [2] and PSO–Simulated Annealing [3] have shown promising results in the fog environments, but they still struggle with the dynamic workload variations and maintaining diversity in them.

Motivated by these findings, this study proposes a hybrid BCO-PSO algorithm for task scheduling in a fog computing environment, and it is focused on balancing the exploration behaviour and exploitation nature to of the respective algorithms to improve makespan and uniform load distribution. This approach follows the recent trend of using hybrid swarm intelligence-based methods to overcome these optimization challenges in dynamic fog environments.

### 1.2. Research question

The above research motivation leads to the following research question:

How can a hybrid Bacterial Colony Optimization and Particle Swarm Optimization (BCO-PSO) algorithm improve load balancing in fog computing with respect to minimizing makespan? How does its performance compare to standalone and other hybrid metaheuristic algorithms across different fog node configurations?

This study is organised as follows. Section 2 shows the related work on fog computing challenges and load balancing needs, metaheuristic algorithms, and hybrid methods. While Section 3 discusses the methodology that includes research strategy, tools, configurations, and evaluation metrics. Section 4 shows the flow diagram and design of the proposed hybrid BCO-PSO algorithm, and Section 5 covers its implementation and outputs. Section 6 presents the performance evaluation on the makespan, average VM utilization and execution time, and a discussion. Lastly, Section 7 concludes with key findings and future directions.

## 2. Related work

### 2.1. Fog computing: Challenges and load balancing needs

Fog computing moves the computation and storage closer to IoT devices through a layered, geographically distributed nodes, such as gateways and routers, that reduce the latency and network bottlenecks [6]. Managing these resources is very challenging due to the differences in node capacity, unstable connections, and real-time processing requirements [7]. Allocation strategies must consider resource capacity, QoS requirements, and unpredictable workloads.

Fog systems must provide low latency with location awareness features and mobility support, which is requiring a flexible load balancing technique to adapt to shifting workloads and network conditions [8]. Without effective balancing, even a minor delay or imbalance can reduce the entire system’s reliability. Many applications have strict deadlines, where poor distribution often causes bottlenecks, idle nodes or missed deadline condition. Smart offloading is important for handling the dynamic workloads efficiently, as poor scheduling leads to latency and energy use in domains such as industrial automation and smart cities [9]. Uneven task distribution can also lower service availability and it reflects the need for better adaptive scheduling [10].

### 2.2. Metaheuristic algorithms in fog environments

Metaheuristic algorithms are those algorithms which is inspired by nature and the evolution process, and they are widely explored in the fog computing domain for solving the complex optimisation problem exhaustive search [11]. They adapt to the changing conditions unlike the traditional methods and could perform better with changing workloads and flexible network condition. This makes them a better alternative solution for fog environment-related issues.

For example, metaheuristic algorithms like Particle Swarm Optimisation (PSO), Ant Colony Optimisation (ACO) and Genetic Algorithms (GA) are considered good for use cases. ACO algorithm is better managing the dynamic task offloading in the fog environments via reducing delays and improving the performance when compared to other methods Hussein and Mousa (2020). Similarly, PSO has achieved a better response time and higher resource utilisation than traditional approaches in fog–cloud [9]. For energy efficiency, a fuzzy logic–driven metaheuristic approach showed much better workloads and resource availability in real time and reduced both latency and energy consumption [12]. So, these studies show that metaheuristic methods can outperform the existing standalone scheduling techniques and can help fog systems to maintain performance under changing conditions.

### 2.3. Particle Swarm Optimisation (PSO): Strengths and drawbacks

The particle swarm optimization (PSO) algorithm is a popular metaheuristic algorithm for load balancing and resource allocation in fog and cloud environments due to its simple and scalable nature and better adaptability [13]. This is inspired by the flocking behaviour of birds or school of fish as PSO represents solutions as particles that update their position depending on their personal best and their neighbourhood experience. This feature provides a much better exploration and exploitation.

PSO algorithm is considered as the alternative for the lower computational overhead that the Genetic Algorithms (GA) or Ant Colony Optimization (ACO) [14]. In a fog computing environment, it has reduced the response time and high throughput by the help of Enhanced Dynamic Resource Allocation (EDRA) [15]. The hybridization method also helped it to enhance its capacity like in PSO–Simulated Annealing for reducing makespan and energy use [3], or PSO-Firefly for the better load distribution [2]. The Bacterial Foraging–PSO is also known for its faster convergence in the optimization tasks [16]. This algorithm has also showed adaptability to the changing infrastructure in dynamic load as discussed by [17].

The PSO also have a weakness of a premature convergence problem in a dynamic fog network [13], and it is sensitive to the parameter settings such as inertia weight and acceleration coefficients [18]. Effective hybridization and parameter tuning are crucial for its performance in fog load balancing.

Some of the other recent works based on multi-objective function and metaheuristic algorithms to improve energy efficiency, resource Utilization, delay, access time, and deadline failure. In [19] authors have proposed an energy-aware load-balancing algorithm for fog computing. The work has proposed a PSO-based energy-aware algorithm to improve the performance in fog computing. In another similar work [20], the author has proposed a multi-objective approach using the marine predator’s algorithm with the polynomial mutation mechanism for scheduling in fog. The work aims to improve the CPU utilization, makespan and energy at the same time, taking into consideration the makespan, CO2 emission and energy consumption into consideration. The work is important because it merges the logic of 2 algorithms to improve the performance. Another similar work [21] where the author has proposed a multi-objective simulated annealing (MOSA) approach for fog to improve access delay and deadline failure into consideration. The work used the same two variables in the fitness function, where the work is compared with the multi-objective moth flame algorithms, Tabu Search and PSO algorithms to study the performance. Another work that uses a hybrid evolutionary algorithm is proposed [22] for energy efficiency using makespan and energy efficiency as fitness functions. The work outperforms PSO and the traditional genetic algorithm. In [23] R. Shahidani has proposed a multi-objective load balancing algorithm using a reinforcement learning algorithm-driven approach to improve the QoS in fog environment. The work aims to improve the makespan and average response time at the same time. The work has been compared with GA, PSO and DRAM to prove the improvement in the fog environment. These existing works uses single optimization algorithm, which may not lead to the best results. There exist many similar works using various combinations of objectives like CPU, CO2, energy, makespan, delay, and many more using various nature inspired algorithm and many other optimization algorithms. This work aims to solve the problem using BCO and PSO.

### 2.4. Bacterial Colony Optimization (BCO): Applicability and gaps

Bacterial Colony Optimization (BCO) was introduced by Niu and Wang [4] which is inspired by the collective foraging behaviour of *E. coli* bacteria colonies. While Bacterial Foraging Optimization (BFO) which mimic only an individual behaviour of bacteria and lacks inter-agent communication, which could lead to slower convergence, BCO, on the other hand, has social cooperation by implementing chemotaxis, communication, elimination, reproduction and migration with a dynamic step size to balance exploration and exploitation [16].

It is a swarm-based algorithm with a diversity containing system that makes a BCO more suitable for multimodal and non-linear optimisation in changing environments. While it is largely applied outside fog computing, it has shown potential of having a strong performance in high dimensional clustering [24] and when it is hybridised with PSO, it achieved a better intra-cluster compactness and inter-cluster separation by combining long-term stability with fast convergence [5].

Even with these strengths BCO is highly underexplored for fog computing implementation, such as load balancing, energy-efficient scheduling and latency. Due to its dynamic search capabilities and potential for giving better results on hybridization makes it is a promising option for improving load balancing.

### 2.5. Hybrid metaheuristics for load balancing in fog

This section summarises hybrid load balancing approaches in fog computing and highlights the motivation for selecting BCO–PSO in this study. Hybrid metaheuristics approach combines two or more optimisation algorithms to overcome the existing limitations of standalone methods. In fog computing, where workloads changes quickly and resources are very constrained these such combinations can merge the fast convergence of one algorithm with the strong search ability of another for more effective task scheduling.

In fog and IoT–fog environment, a HybOff approach was used that had static–dynamic offloading with the clustering method to reduce decision overhead and gave shorter offloading distance with improved utilisation [1]. Harris Hawks–MothFlame Optimisation reduced the latency and showed high energy efficiency [25], while PSO–Firefly improved the load distribution and gave much lowers makespan. PSO–ACO hybrids method, on the other hand also showed that the cost and scheduling could be improved when compared to their old versions [26]. The IABC–MBOA hybrids was developed for cloud environments but could be adapted to fog as well [27].

Relevant to this study, the Bacterial Foraging–PSO algorithm achieved a faster and more stable convergence [16] and BCO–PSO hybrids improved the clustering accuracy by combining BCO’s search diversity with PSO’s convergence speed [5]. Some other PSO-based hybrids such as PSO–Simulated Annealing and NDWPSO (PSO with Differential Evolution and Whale Optimisation) [28], addressed the premature convergence problem and improved its quality.

## 3. Methodology

### 3.1. Research strategy

Task scheduling in fog computing is an NP-hard optimisation problem [29] in which the existing methods often fail to adapt to the various workload variations and changing node capabilities. This study proposes a hybrid Bacterial Colony Optimization–Particle Swarm Optimization (BCO-PSO) approach to combine the fast convergence of PSO [13] with the stable exploration capability of BCO [4] and considers the Adaptive Inertia Weight PSO (AIW–PSO) for comparison benchmark [30]. The objective is to achieve better load balancing by minimizing the makespan while improving the resource utilisation in fog environments. This study follows an offline (batch) scheduling model where all tasks are known a priori.

### 3.2. Tools and environment setup

All the experiments were implemented in the Python 3.10 using the MEALPY library (v3.0.1) [31] for the standard PSO baseline. The BCO and hybrid BCO-PSO algorithms were manually implemented to allow precise control over chemotaxis, reproduction, and swarm updates. The implementation and execution were done on Google Colab platform, which provided the required computational resources for the algorithms’ testing. Data processing, logging and visualisation used NumPy and Matplotlib. Workload datasets were from synthetic JSON files; full details are provided in Section 3.6. The algorithms and synthetic datasets used in this study are hosted in a GitHub repository and are available from the authors upon reasonable request to support reproducibility.

### 3.3. Fitness function

The objective in this study is *makespan minimisation,* which is a standard metric in fog computing scheduling that measures the maximum completion time among all virtual machines (VMs) after task allocation. A smaller makespan value indicates a more even distribution of workloads and better utilisation of resources. Formally, let:

• M denote the number of virtual machines (VMs),

• $T_{j}$ denote the set of tasks assigned to VM j,

• ${\text{TaskLoad}}_{i}$ denote the CPU load required by task i,

• ${\text{VMCapacity}}_{j}$ denote the processing capacity of VM j.

The makespan is calculated as:


$$\text{Makespan}=\underset{j=\text{1},\ldots ,M}{\text{max}}\left(\frac{\sum_{i\in T_{j}}{\text{TaskLoad}}_{i}}{{\text{VMCapacity}}_{j}}\right)$$

where the term $\frac{\sum_{i\in T_{j}}{\text{TaskLoad}}_{i}}{{\text{VMCapacity}}_{j}}$ represents the execution time of VM j, and the maximum operator selects the most heavily loaded VM.

In addition to makespan, *average VM utilisation* is used as a secondary performance metric to evaluate how evenly the workload is distributed across available virtual machines. Average VM utilisation is defined as the mean normalised load across all VMs and is calculated as:


$$\text{Average VM Utilisation}=\frac{\text{1}}{M}\sum_{j=\text{1}}^{M}\left(\frac{\sum_{i\in T_{j}}{\text{TaskLoad}}_{i}}{{\text{VMCapacity}}_{j}}\right)$$

Higher average VM utilisation indicates better load balancing across the fog infrastructure. In the implementation, the makespan objective is evaluated within the fun() function during optimisation. After the optimisation process, the fun1() function is used for final reporting of both makespan and average VM utilisation based on the best solution obtained. This study adopts a single-objective optimisation strategy where makespan is prioritised as the primary scheduling objective, while other metrics are evaluated separately.

### 3.4. Research methodology workflow

**Fig 2:**
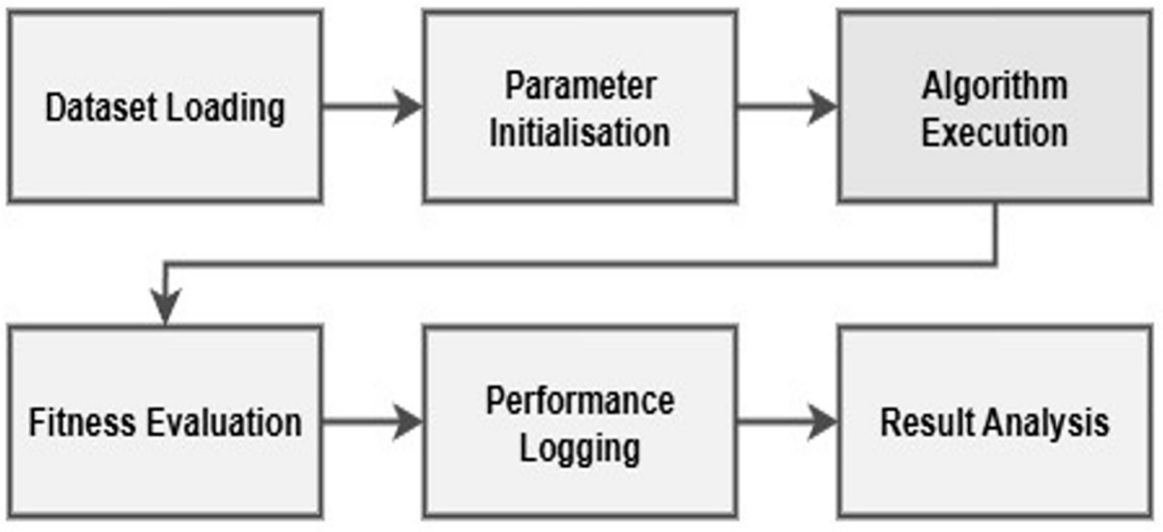
Research methodology workflow for algorithm evaluation.

The experimental workflow followed in this study is shown in Fig 2:

1. **Dataset Loading:** We load the JSON dataset that contains task CPU load and number of VM required.
2. **Parameter Initialisation:** Set the algorithm parameters like the population size, iteration or the step sizes as required.
3. **Algorithm Execution:** Run all four BCO, PSO, AIW-PSO, and Hybrid BCOPSO algorithms on the same dataset given.
4. **Fitness Evaluation:** Calculate the makespan for each cases using the Equation 3.3.
5. **Performance Logging:** Save all the best fitness, execution time, and VM utilisation results to a JSON file.
6. **Result Analysis:** Generates convergence curves and performance charts using results.

### 3.5. Hybrid algorithm implementation

The hybrid BCO–PSO algorithm, as described in Section 4.4 was executed alongside with standalone BCO, standard PSO, and the Adaptive Inertia Weight Particle Swarm Optimization (AIW–PSO) to provide a fair performance comparison. The AIW–PSO variant Nickabadi et al. (2011) was selected as a representative adaptive PSO algorithm that introduced a dynamically adjusted inertia weight to improve the balance between exploration and exploitation. For all experiments, the hybrid used the parameter settings listed in Table 1 and operated on the same JSON task–VM datasets. The experiment applied the algorithm to assign tasks to 10 heterogeneous VMs in a simulated fog environment with makespan as the optimisation objective.

**Table 1:** Experimental parameter settings.

Parameter	Value/ Description
Number of Tasks	100, 200, 1000, 5000, 10,000
Number of VMs	10 (Fixed)
Fitness Function	Makespan minimisation
Execution Platform	Google Colab (Python 3.10)
Library Used	MEALPY v3.0.1
**Common Parameters**	
Population Size	100 agents
Iterations	50
**BCO Parameters**	
$\frac{StepSizeRange}{ChemotaxisPower}$ $\frac{C_{min}=\text{0}.\text{1},C_{max}=\text{0}.\text{1}}{n=\text{2}}$	
Reproduction Interval	20 iterations
**PSO Parameters**	
Inertia Weight (*w*)	0.5
Cognitive Coefficient (*c*_1_)	1.5
Social Coefficient (*c*_2_)	1.5
**Hybrid BCO–PSO Parameters**	
PSO Update Interval	Every 30 iterations
PSO Update Probability	10% per bacterium
PSO Parameters (Hybrid step)	*w* = 0.5*, c*_1_ = 1.5*, c*_2_ = 1.5

*Fitness function implementation.* The same Gaussian perturbation technique described in Section 3.3 was applied to each task’s VM index before rounding to promote exploration while preserving discrete assignments. In addition, the chemotaxis step size *C* was adjusted adaptively at each iteration, decreasing from *C*_max_ to *C*_min_ based on the iteration count, enabling broader exploration early on and finer exploitation later. To maintain complete fairness all algorithms were executed under the same parameter settings and dataset configurations with random initialisations for each run.

### 3.6. Experimental design

The evaluation process compares the proposed hybrid BCO–PSO against the standalone BCO, standard PSO and AIW–PSO using identical datasets, fitness functions, and execution parameters to maintain fairness. The dataset comprises tasks (with CPU load and execution time attributes) mapped to a fixed set of 10 virtual machines (VMs) with predefined processing capacities. Dataset of various sizes tested were 100, 200,1000, 5000 and 10,000 tasks, which provided scalable analysis. The full simulation hardware and VM configuration is detailed in Section 3.7.

### 3.7. Simulation configuration

The experiments were conducted in a simulated heterogeneous environment composed of a cloud data centre and three edge server types. Each of the workload scenarios used 10 VMs with tasks assigned in five configurations: 100, 200, 1000, 5000, and 10000 tasks. This range allowed evaluation under light, medium, and heavy loads. The specifications of the simulated servers are provided in Table 2.

**Table 2:** Configuration of cloud and edge servers.

	Cloud Datacentre	Server Type 1	Server Type 2	Server Type 3
Core	200	5	4	8
MIPS	40000	20000	4000	10000
RAM	16000	8000	4000	4000
Storage	1000000	1024000	32000	128000
Bandwidth (MB)	10000	5000	4000	5000

This setup mimics a fog computing deployment where edge nodes differ in processing capacity and memory. It makes sure that algorithms are tested under resource conditions that make the evaluation process more representative.

**Metrics.** We evaluated the algorithms using three key metrics:

- **Makespan** – The maximum load on any single VM. The lower the number, the better the balance.
- **Average VM Utilisation** – The average (normalised) load across all VMs. Closer to equal means more evenly used.
- **Execution Time** – How long the algorithm takes to run.

**Procedure.** Each algorithm was executed on all dataset sizes using the same fitness function and parameters. Outputs include final metrics, per-iteration best fitness values, convergence plots, and JSON task–VM assignments were logged for reproducibility and analysis purposes.

### 3.8. Data collection and analysis

Raw performance data from each run, which included the per-iteration best fitness values and final makespan, execution time, and VM utilisation metrics, were automatically saved to a JSON results file after each execution. Each new run appended its metrics to this file, which provides a cumulative storage of results for algorithm dataset combinations. This provides reproducibility and helps in tracking independent runs for each experimental scenario and capturing the natural variability in stochastic optimisation.

The mean, best, and standard deviation were calculated for makespan, execution time, and VM utilisation with all algorithms evaluated using identical datasets, parameters, and stopping criteria. Analysis and visualisation were performed in Python using NumPy for numerical aggregation and Matplotlib for plotting convergence curves, and bar charts were generated separately during the results visualisation stage. Algorithm performance was compared using average metrics, following common practice in fog computing optimisation studies.

## 4. Design specification

### 4.1. System overview

The proposed system is designed to perform task scheduling in a heterogeneous fog computing environment by using a hybrid BCO–PSO algorithm. It uses the JSON dataset which provides the computational tasks and the capacities of virtual machines (VMs). The scheduling process is focused on minimise the makespan by distributing the workload evenly across the VMs. The architecture consists of a dataset loader, an optimisation engine implementing the selected scheduling strategy (BCO, PSO, AIW–PSO, and hybrid), and then an evaluation module for calculating the performance metrics. The optimisation engine updates task–VM assignments, while the evaluation module records results for later analysis and comparison.

### 4.2. Requirements and assumptions

The system operates under the following:

- Input dataset is in JSON format with task CPU loads, execution times, and a fixed set of 10 VMs.
- Tasks are independent with no precedence constraints or deadlines.
- Candidate solutions are real-valued vectors, discretised to VM indices by rounding; ties are resolved by assigning to the lowest-load VM.
- All tasks must be assigned to exactly one VM.
- Network delays and bandwidth are not modelled; optimisation focuses solely on computation load. Therefore, results represent algorithm-level performance rather than a full fog system deployment.
- Tasks are scheduled in an offline (batch) manner and dynamic task arrivals are not considered.
- Parameter settings follow Table 1 in Section 3.

### 4.3. Architecture flow diagram

**Fig 3:**
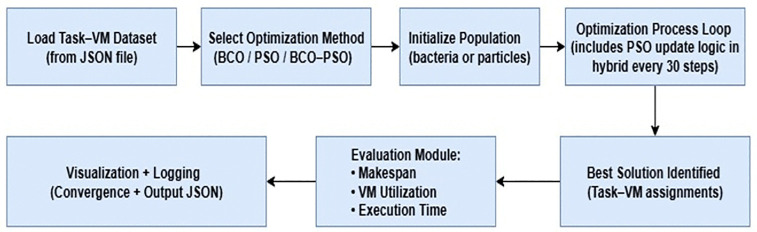
Architecture flow diagram for task scheduling algorithms.

The architecture provides the complete workflow for executing and evaluating in the simulated fog environment. The complete workflow is summarised in Fig 3, showing how the JSON dataset is passed into the optimisation engine and how results are logged and analysed. The process starts with the loading of the data from the JSON dataset, which contains the task CPU requirements and the specifications of the VMs. This data is then passed to the optimisation module in which applies the selected scheduling method. The optimisation loop iteratively updates the candidate task-VM assignments according to the algorithm’s search logic and then records the best fitness values at each step.

On completion, the evaluation module then calculates the overall makespan, average VM utilisation, and execution time based on the final assignment. All outputs include the metrics, convergence data, and task-VM mappings are stored in the JSON log. This structure makes sure that the same dataset with optimisation process and evaluation are applied for comparison across all tested algorithms.

### 4.4. Proposed hybrid BCO–PSO algorithm

The proposed hybrid BCO-PSO algorithm integrates the adaptive chemotaxis behaviour of Bacterial Colony Optimization (BCO) with the global search capability of Particle Swarm Optimization (PSO) to improve the task scheduling in heterogeneous fog environments. The design is motivated by the complementary strengths of the two algorithms: BCO, which is good at fine grained local exploration and PSO, which accelerates the convergence towards high-quality global optima.

**Fig 4:**
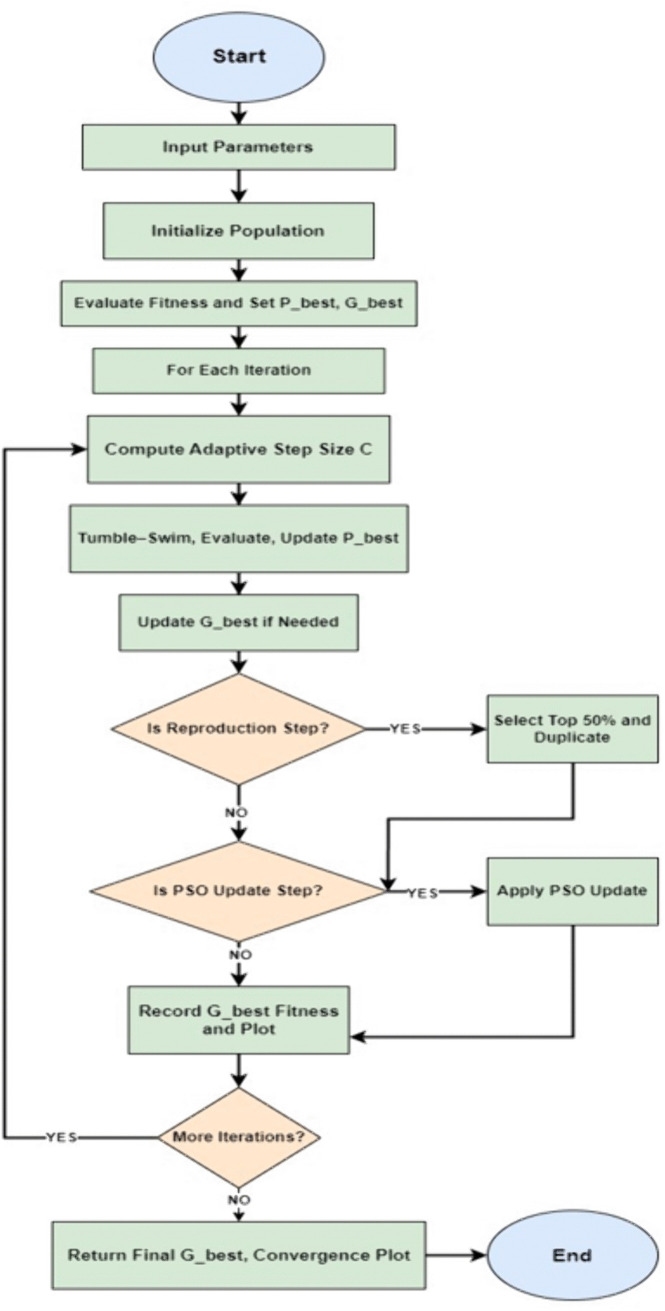
Flowchart of the proposed hybrid BCO-PSO algorithm.

The step-by-step logic of the hybrid approach is illustrated in Fig 4, which shows how the BCO chemotaxis–reproduction cycle is combined with periodic PSO updates. The algorithm begins by randomly initialising a population of bacteria (candidate task-VM assignments) within the VM index bounds. Each bacterium represents a complete schedule and VM assignments. Fitness is then evaluated using the makespan objective, in which the lower values indicating better load balancing.

In each iteration, bacteria perform a chemotaxis process using the adaptive tumble swim strategy where the step size *C* decreases over the iterations to balance exploration in early stages and exploitation in later stages. Candidate positions are then updated based on the current orientation, which is modified by a turbulence factor to maintain the diversity and avoid premature convergence.

Reproduction occurs at every 20 iterations, where the top 50% of bacteria (based on fitness) are duplicated to replace the bottom 50%; this process preserves the best performing schedules while maintaining the population size. Personal best (*P*_best_) values are tracked for each bacterium, and the global best (*G*_best_) is updated when improvements are found.

Hybridisation with PSO is applied every 30 iterations but only with a small probability (as per parameters in Table 1) to avoid over-dominating the BCO workflow. In this step, velocities are updated according to the standard PSO equation by combining the inertia, cognitive and social components, and then it is applied to bacterial positions. This targeted PSO injection improves the ability to escape from local optima without sacrificing BCO’s adaptability nature. All updated positions are kept within the VM limits and converted to valid VM indices by rounding off and then evaluated again. The best solution from all iterations is returned with the convergence for analysis.

**Algorithm 1.** **Hybrid BCO-PSO algorithm.**

Input: Task-VM bounds, number of bacteria, max iterations, BCO and PSO parameters

Initialize population positions and velocities randomly

Evaluate fitness for all individuals

Set personal bests (P best) and global best (G best) for each iteration:

Compute adaptive step size C for each bacterium:

Move using BCO tumble-swim logic

Evaluate new fitness

If improved, update current position and fitness

If fitness better than P best, update P best

Update G _best if needed if at reproduction step:

Select top 50% bacteria and duplicate them if at PSO update step:

For each bacterium, with small probability:

Update velocity using PSO formula

Update position and re-evaluate fitness

Record G best fitness at each iteration

Return: Final G best, best score, convergence plot

**Computational Complexity.** Let N be the population size and I be the number of iterations. Since fitness is evaluated for N solutions in each iteration so the overall runtime is approximately O(N×I). The periodic PSO update is applied to only a subset of solutions, so it does not change the overall order.

### 4.5. Design justification

The hybrid design was chosen to overcome the weaknesses of using standalone BCO or PSO for fog-based scheduling. BCO’s adaptive tumble-swim nature is good for exploring the nearby solutions in large search spaces Niu and Wang (2012), but it can get stuck without a way to find new promising areas. While PSO can quickly guide the search towards a global best solution Freitas et al. (2020), it sometimes converges too early in the process. Our approach adds PSO updates only at set intervals and with low probability so that BCO could keep its local search strength while PSO helps it to escape from local optima. This combination improves convergence speed, avoids stagnation and is better suited for the fast-decision-making process that is needed in a time-constrained scheduling scenario.

## 5. Implementation

### 5.1. Development of the simulation model

This section shows the final implementation of the proposed hybrid BCO-PSO algorithm for load balancing in fog computing environments. The implementation carries the simulation of task scheduling across the virtual machines (VMs) using metaheuristic optimization techniques. All experiments were performed on the Google Colab platform using Python 3.10. The BCO and Hybrid BCO-PSO algorithms were custom-developed in Python, while the standard PSO and AIW–PSO algorithms were executed using the MEALPY v3.0.1 library.

The final system takes as input a JSON-formatted dataset defining task loads and VM capacities, processes the data using the selected scheduling algorithm and outputs a task-to-VM assignment optimized to minimize makespan. This implementation then simulates the real fog conditions with varying workloads and resource configurations, tested on synthetic datasets of 100, 200,1,000, 5,000 and 10,000 tasks generated by CloudSim 6.0 which allows to setup EDGE environment using poison distribution of task requests. The configuration of the simulation environment, tools used and algorithmic parameters are detailed below, followed by the outputs generated during evaluation.

### 5.2. Tools and technologies used

- **Google Colab:** A cloud-based Jupyter notebook environment was used to run all simulations and visualizations of algorithms.
- **Python 3.10:** The main programming language for algorithm development and simulation execution for the project.
- **MEALPY v3.0.1:** A metaheuristic optimization library that is used to run the PSO and AIW-PSO algorithms for baseline comparison with a hybrid algorithm.
- **Supporting Libraries:** numpy (numerical operations), json (dataset parsing), random (stochastic updates), time (execution tracking), and matplotlib (visualizations).

### 5.3. Execution workflow

In the final stage, the complete simulation environment was set up in such a way that each experiment could run from dataset loading to result visualisation without any manual interruption. This confirms that algorithms were tested under the same conditions and avoid any bias. The workflow was as follows:

1. The list of tasks is generated by Cloudsim 6.0 using the simulation configuration of fog, edge and cloud datacenters and host.
2. The simulation notebook in Google Colab firstly loads the specified JSON dataset (e.g., data _1000 10.json) generated in previous step from the mounted directory and checks that the task and VM definitions match the expected format.
3. The optimization module receives the dataset and runs the selected algorithm using the same settings for population size, number of iterations, and step size.
4. The loop runs for the set number of iterations. In the Hybrid BCO-PSO case, PSO velocity and position updates are injected at the configured intervals within the BCO chemotaxis–reproduction cycle.
5. At the end of execution, the final task-VM allocation, calculated makespan, average VM utilisation, and iteration-wise best fitness values are saved into a structured JSON results file (output _final comparison.json).
6. Convergence curves were generated automatically by using the stored results while the comparative bar charts were created manually using the exported metrics to provide consistent formatting for all algorithms.
7. The results are then input to cloudsim simulator for final results and performance evaluation.

By keeping the workflow under the same conditions across the separate Google Colab notebooks for each algorithm, every run follows the same sequence and settings, which ensures that the evaluation was consistent, reproducible, and free from manual bias.

### 5.4. Outputs produced

The simulation produced the following key outputs:

- **Task–VM Assignment:** The computed mapping of tasks to VMs for each algorithm, optimised to minimise makespan while avoiding overload on any single VM.
- **Performance Metrics:** Recorded values for makespan, average VM utilisation (in %), and total execution time for each experimental run.
- **Convergence Curves:** Iteration-wise plots of the best fitness value, that is showing the speed and stability of convergence.
- **Result Logging:** Structured JSON file (output final comparison.json) does all the storage of metrics and mappings for the post processing and comparison to take place.

These outputs form the basis of the evaluation presented in the next section, enabling a direct performance comparison of BCO, PSO, AIW–PSO, and the proposed Hybrid BCO–PSO.

## 6. Evaluation

This section evaluates the proposed Hybrid BCO–PSO algorithm for load balancing in fog computing environments through comparing it with the standalone BCO, standard PSO, and AIW–PSO is also included as a known swarm-based scheduling benchmark. The analysis focuses on the three critical performance metrics: makespan, execution time, Energy and average VM utilisation. These metrics are directly responsible for making impact on the scheduling efficiency, system response time and resource usage in fog computing. Therefore, it provides a clear basis for assessing whether the proposed hybrid approach achieves its targeted performance improvements. All these experiments were carried out in a Python-based simulation using MEALPY v3.0.1 in Google Colab with task–VM datasets of varying sizes under a simulated fog environment.

### 6.1. Simulation setup with makespan as fitness function

#### i. Performance Metric 1: Makespan.

Makespan is the total time taken to complete all the tasks in a workload from starting to finish. It is an important measure for the load balancing scenarios because it shows how well the tasks are distributed across the available resources. A lower makespan means that tasks are spread out efficiently and avoids delay and finishes the work in less amount of time.

**Fig 5:**
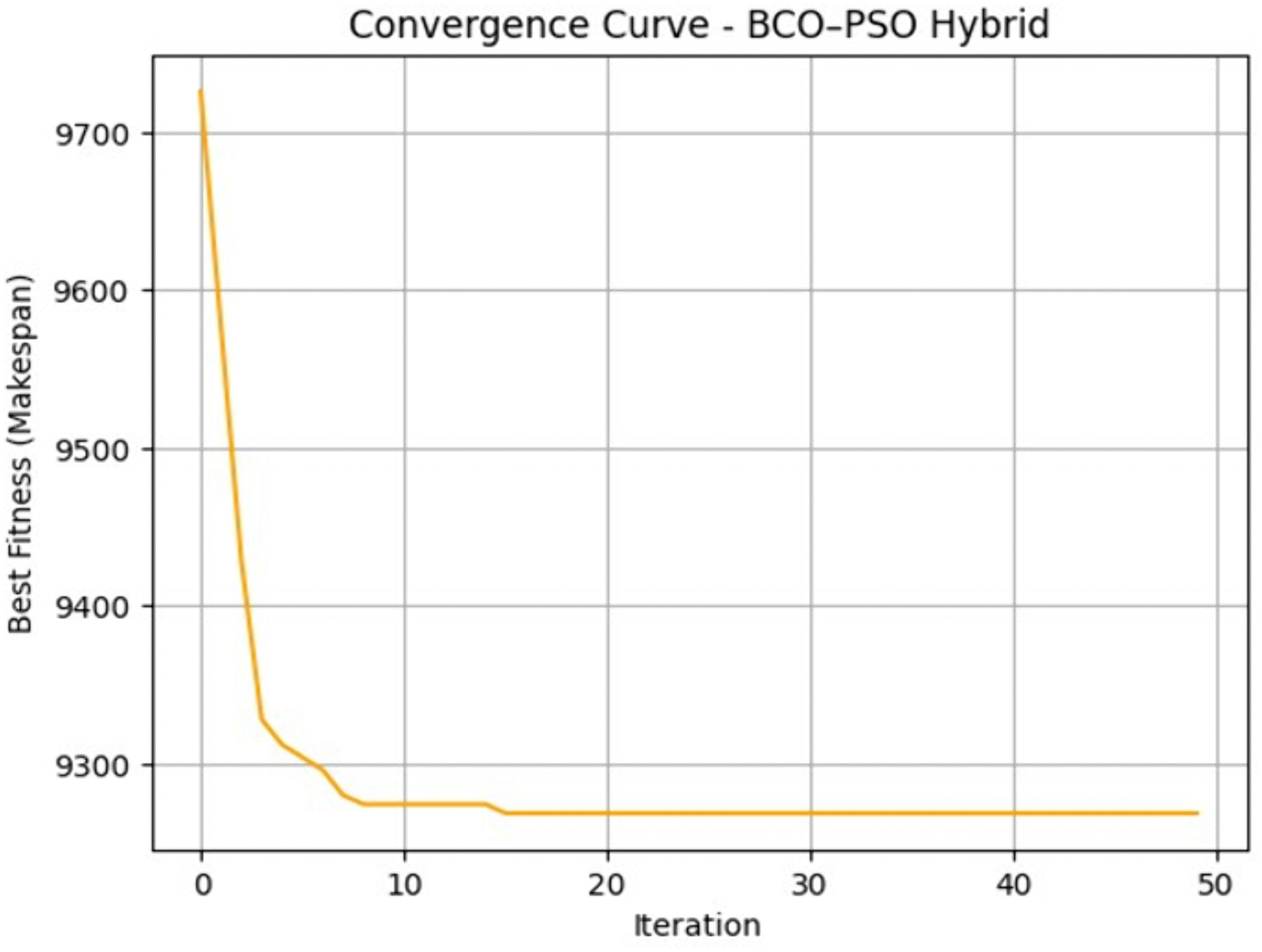
Best makespan comparison across different workloads (100–10000 tasks, 10 VMs) for Hybrid BCO–PSO, PSO, AIW–PSO, and BCO.

**Interpretation.** The proposed Hybrid BCO-PSO consistently achieved the lower makespan in almost all the workloads. For smaller workloads (100 and 200 tasks) the performance was similar to PSO. This indicates that the hybrid’s advantages are most significant as the task volume and the search space was increased. In larger workloads, improvements were more significant. for example, with 5000 tasks, Hybrid BCO-PSO achieved a makespan of 4616.00 compared to the AIW-PSO’s 6864.00, a 32.76% reduction. For 10000 tasks, it reduced makespan by about 0.30% over PSO and 35.8% over AIW-PSO (AIW–PSO originally proposed by Nickabadi et al. (2011)). These improvements come from combining BCO’s exploration process which helps to avoid getting stuck on poor solutions too early, and with the PSO’s speed-focused updates that quickly improve good solutions and assign tasks more efficiently. For the light-load case (100 tasks/ 10 VMs), the makespan comparison is shown in Fig 5, where all algorithms exhibit near-identical values, which is consistent with the results reported in Table 3.

**Table 3:** Best Makespan results for different algorithms across workloads.

Tasks/VMs	Hybrid BCO–PSO	PSO	AIW–PSO	BCO
100/10	4.714	4.714	4.714	5.029
200/10	165.71	177.14	177.14	182.86
1000/10	908.57	914.29	954.29	914.29
5000/10	4616.00	4691.43	6864.00	4668.57
10000/10	9268.57	9296.00	14432.00	9337.14

**Fig 6:**
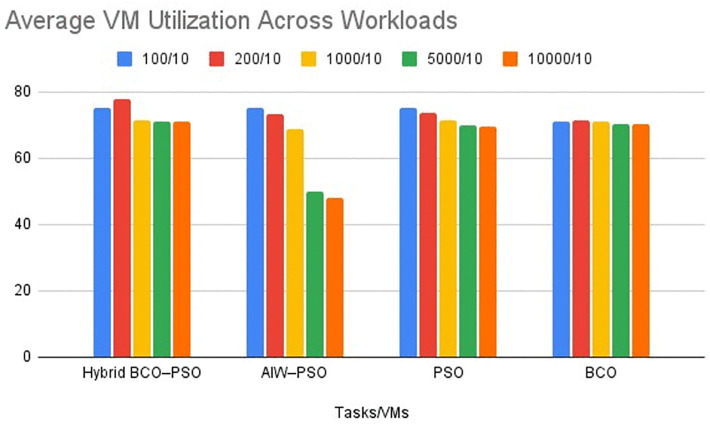
Convergence curve of the Hybrid BCO–PSO for the 10000 tasks/ 10 VMs scenario, showing makespan reduction across iterations.

**Convergence Behaviour.** Fig 6 shows the convergence curve of the Hybrid BCOPSO for the largest workload (10000 tasks/ 10 VMs). The algorithm stabilised near its optimal makespan within 15 iterations which indicates the fast convergence under high-load conditions which is particularly beneficial in time-constrained scheduling scenarios where rapid task allocation is critical. This plot serves as a representative example; additional convergence curves for other workloads are provided in the Configuration Manual.

#### ii. Performance Metric 2: Average VM utilization.

**Fig 7:**
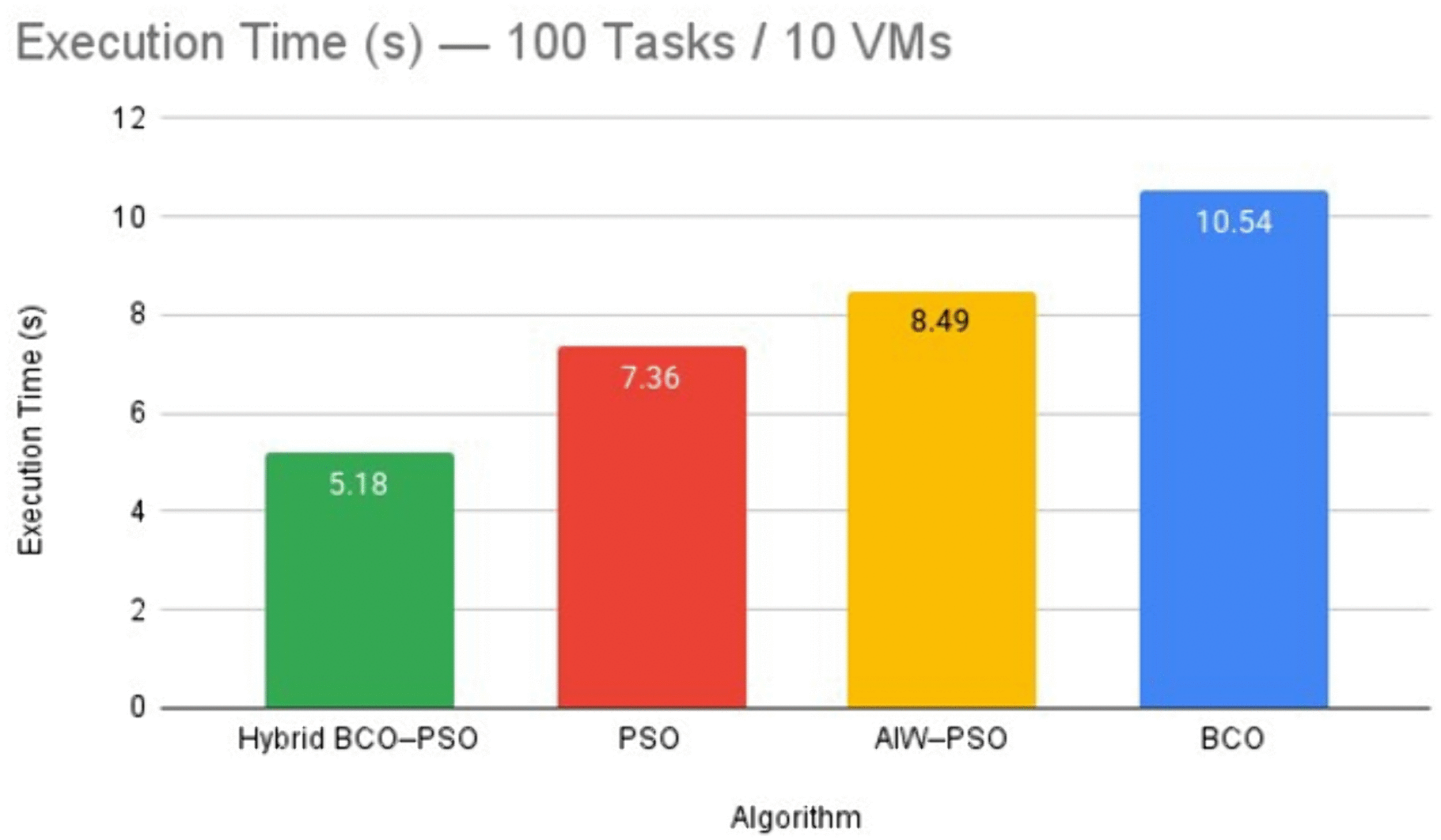
Average VM utilisation comparison across workloads (100–10000 tasks, 10 VMs) for BCO, PSO, AIW–PSO, and Hybrid BCO–PSO.

Average VM utilization measures how effectively available VM resources are used during task execution. The higher utilization shows a better load balancing. The utilisation trend across workloads is visualised in Fig 7, which complements the values reported in Table 4.

**Table 4:** Average VM utilization (%) for different algorithms across workloads.

Tasks/VMs	Hybrid BCO–PSO	AIW–PSO	PSO	BCO
100/10	75.33	75.07	75.33	70.88
200/10	77.93	73.35	73.74	71.31
1000/10	71.40	68.63	71.43	71.09
5000/10	71.17	50.01	69.93	70.30
10000/10	70.90	47.89	69.60	70.26

**Interpretation:** Hybrid BCO–PSO maintained the highest utilization in most cases this peaking at 77.93% for 200 tasks. For smaller workloads (100 and 200 tasks) this utilisation levels were close between Hybrid BCO–PSO and PSO that indicates balanced resource usage even when demand was low. In large workloads, AIW–PSO’s utilization dropped sharply- for example, form 50.01% for 5000 tasks and 47.89% for 10000 tasks. Meanwhile PSO and BCO remained stable but consistently showed below the hybrid model. The hybrid approach’s broader exploration phase and refinement step help it to prevent overloading specific VMs and provide high utilization.

#### iii. Performance Metric 3: Execution time.

Execution time is the time taken by the algorithm to complete scheduling excluding actual task execution on VMs. Lower execution times are beneficial for real-time environments fog environment. The execution time results across all workload sizes are summarised in Table 5.

**Table 5:** Execution time (seconds) for different algorithms across workloads.

Tasks/VMs	Hybrid BCO–PSO	AIW–PSO	PSO	BCO
100/10	5.18	8.49	7.36	10.54
200/10	10.55	9.70	9.99	9.05
1000/10	39.28	38.25	37.66	41.13
5000/10	184.83	263.39	187.07	198.10
10000/10	351.80	472.67	512.70	419.64

**Fig 8:**
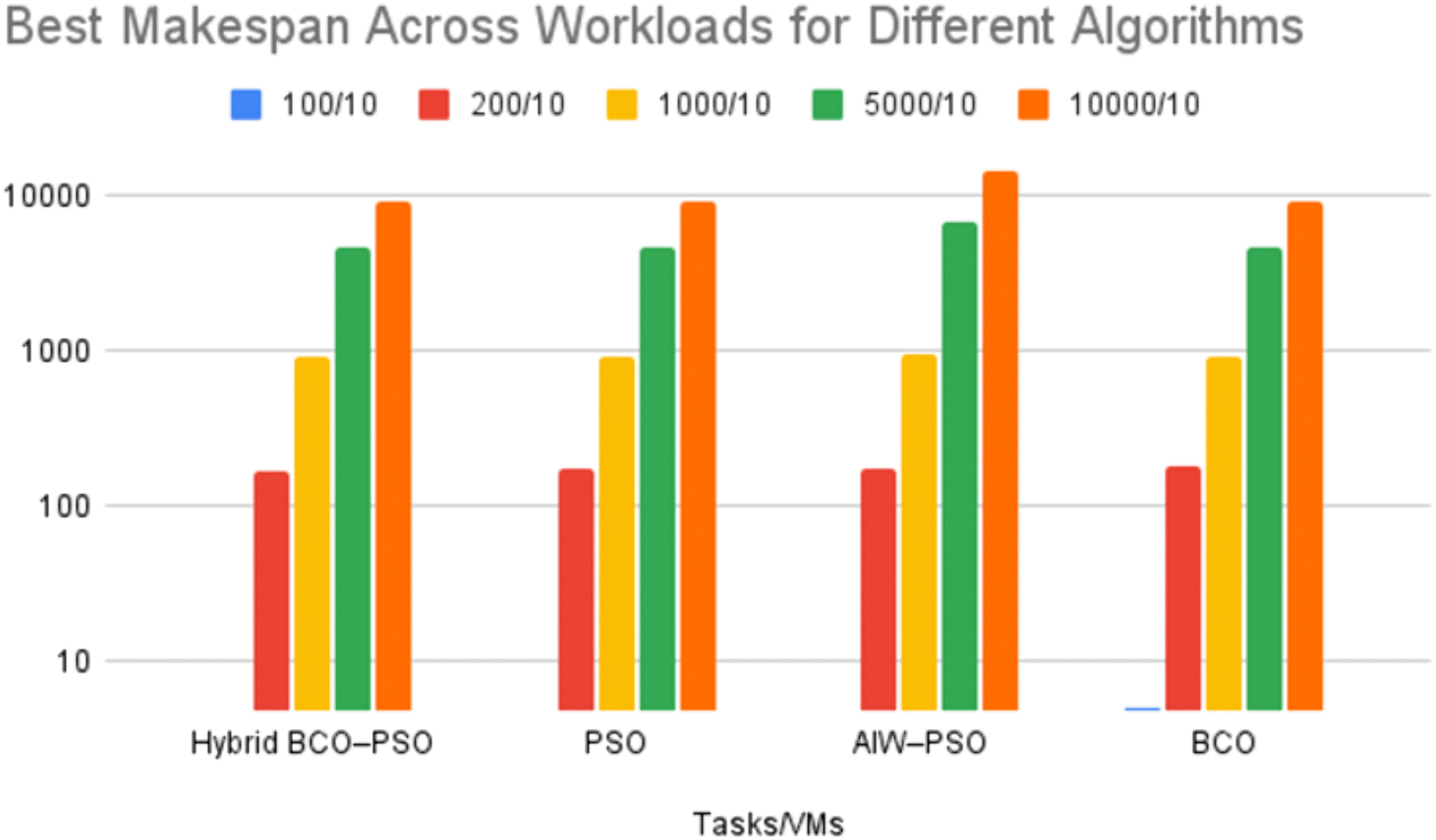
Execution time comparison for the 100 tasks/ 10 VMs scenario across all evaluated algorithms.

**Interpretation.** Execution times varied with workload size in the experiment. Hybrid BCO-PSO was fastest in small workloads (100 tasks) with 5.18 seconds, which benefited from rapid convergence by help of PSO’s velocity updates combined with BCO’s initial exploration that avoided wasted iterations. In large workloads (5000 and 10000 tasks) it again outperformed other methods- for example, completing scheduling in 351.80 seconds for 10000 tasks compared to 472.67 seconds for AIW-PSO because the hybrid algorithm’s periodic refinement steps reduced the number of iterations needed to stabilise the solution. In medium workloads, BCO was fastest at 200 tasks, while PSO was fastest at 1000 tasks due to its simpler update mechanism and lower computational overhead per iteration. However, the hybrid has a slightly higher cost in these cases was offset in larger workloads by better load balancing and faster convergence to high-quality solutions. For a clear example under light load, Fig 8 compares execution time for the 100-task case across all algorithms.

**Fig 9:**
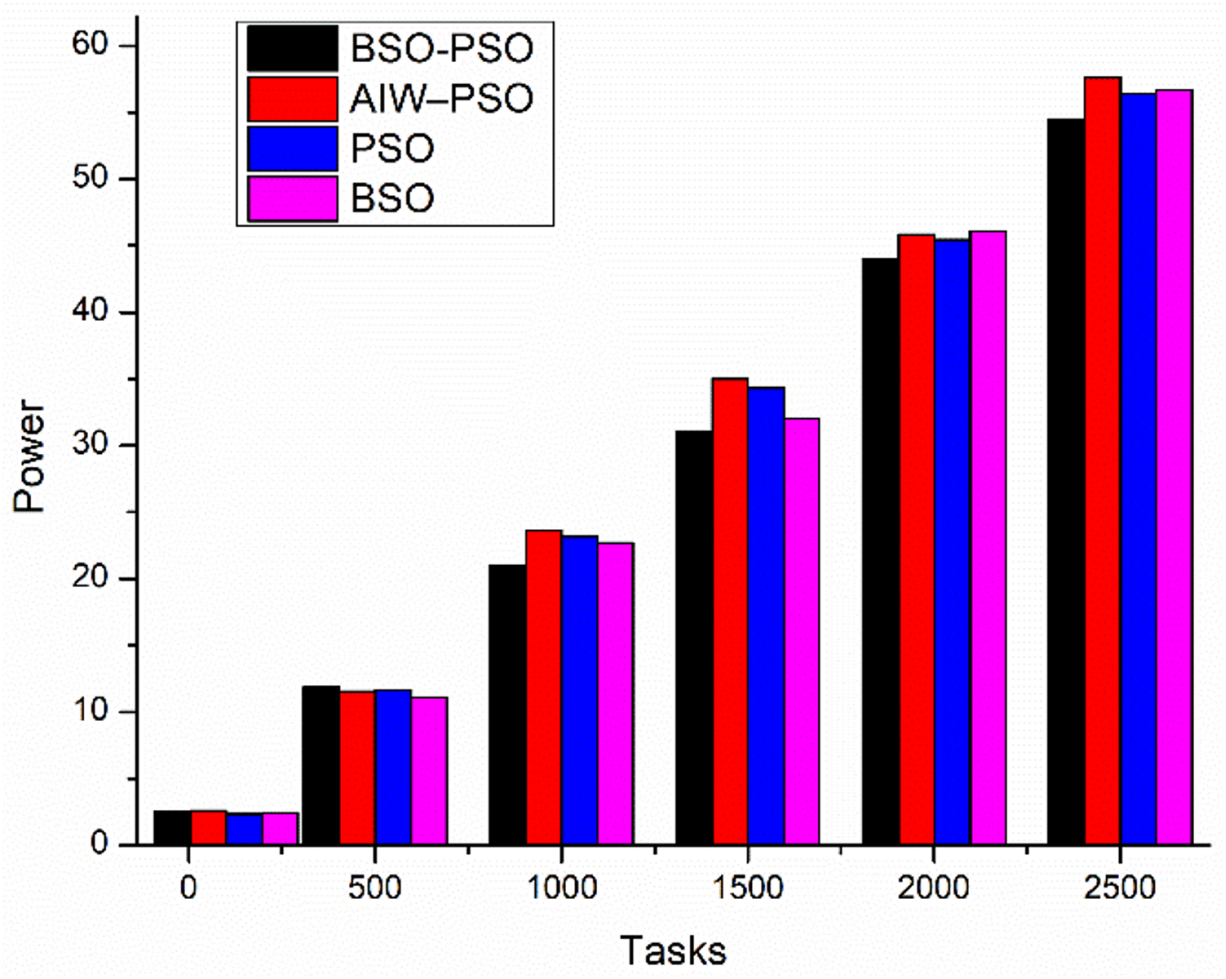
Comparison of Power consumption(kwh) with scaling tasks.

Fig 9 show cases a comparative study of power consumed by VM’s with scaling tasks. The results shows that the proposed algorithm improved the energy consumption with increasing load because with proposed algorithm takes into account the least execution time which result in task landing on a VM with least execution time resulting in less power consumption as compared to existing algorithms.

### 6.2. Simulation setup with utilization as fitness function

In this setup the proposed Hybrid BCO-PSO algorithm is been tested with Utilization as a fitness function. Where proposed algorithm, PSO, AIWPSO and BCO are been texted as utilization as fitness function. Where the fitness is defined as:


$$Fitness(i)=Min({Utilization}_{i})$$

**Fig 10:**
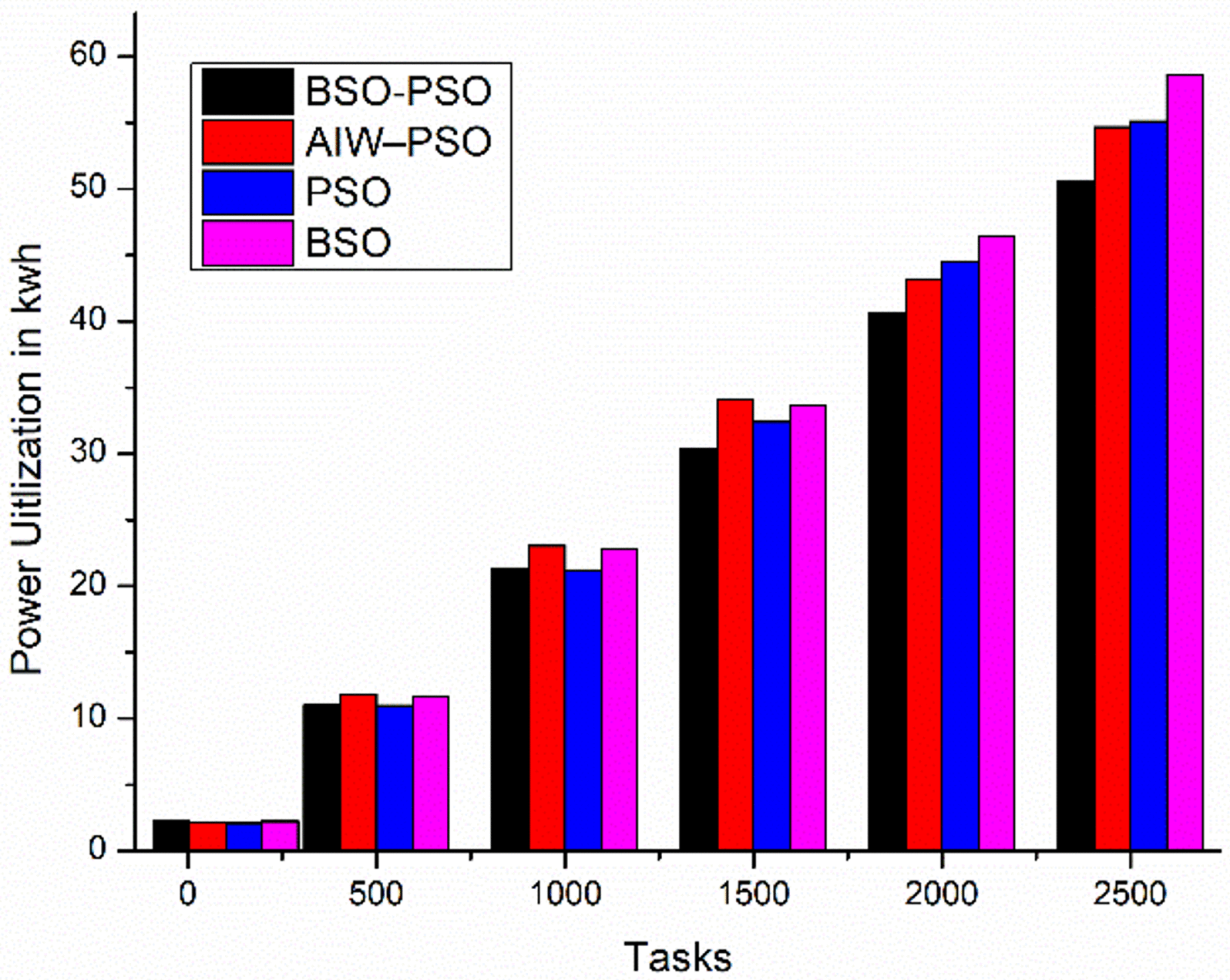
Comparison of Power consumption(kwh).

**Fig 11:**
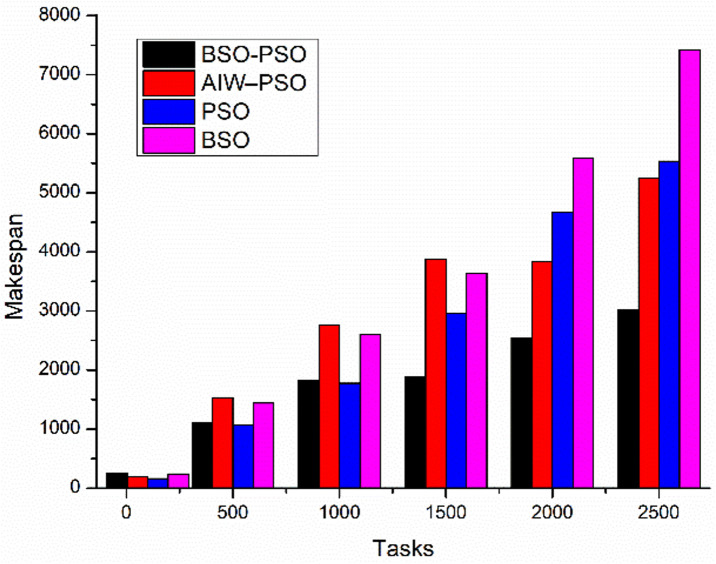
Comparison of Makespan with scaling tasks.

The experiment aims to test the performance of the proposed model under different conditions where the fitness function aims to improve the average utilization. The study also shows the changes in Makespan, finish time and power with change in fitness function. The results are been compared to existing algorithms. Where Fig 10 shows the study of power utilization by the algorithms with increasing number of tasks. The results shows the proposed BSO-PSO overcomes all the exiting models and the improvement in power consumption increases with increasing load. Same with the case of Makespan as shown in Fig 11 the proposed model out performs the existing models with high margin with increasing load where the fitness function with taken as minimum utilization.

**Fig 12:**
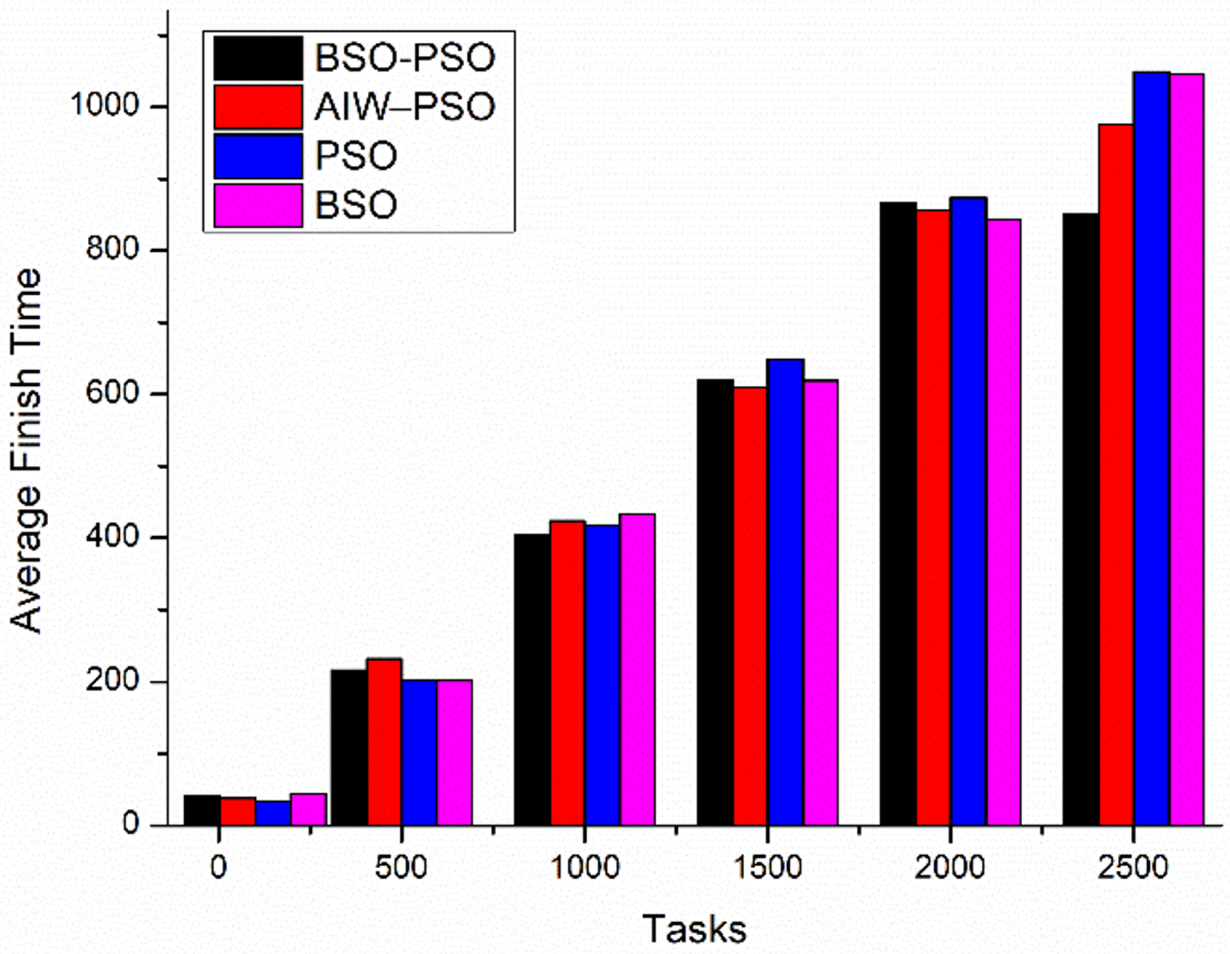
Comparison of Average finish time.

**Fig 13:**
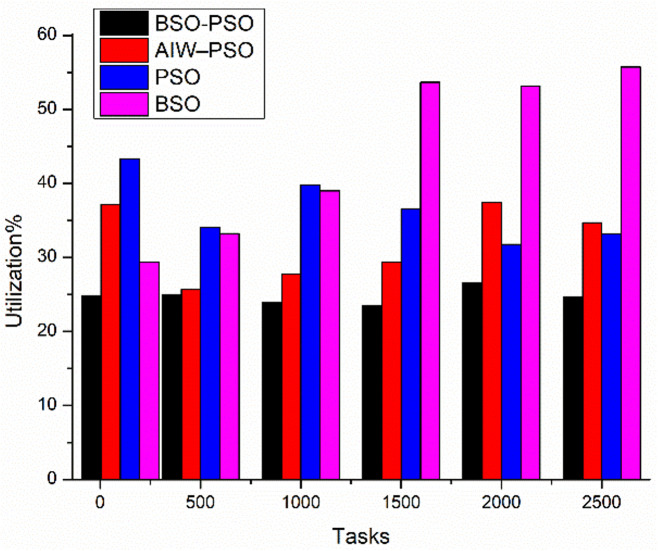
Utilization with scaling tasks.

Fig 12 shows the comparative study of average finish time and Utilization. Where the proposed model improves the average finish time of all tasks as compared to the other models and also the utilization of od resources improves as the load increases (Fig 13).

### 6.3. Simulation setup with multi objective utilization and makespan as fitness function

In this simulation setup the proposed algorithm and other existing algorithms are been tested under multi objective function with two objective function of utilization and power consumption as fitness function. Where the fitness is defined as:


$$Fitness(i)=\text{min}(\alpha {*Utilization}_{i}{+\beta *Makespan}_{i})$$

whereα,βaretakenas0.5

**Fig 14:**
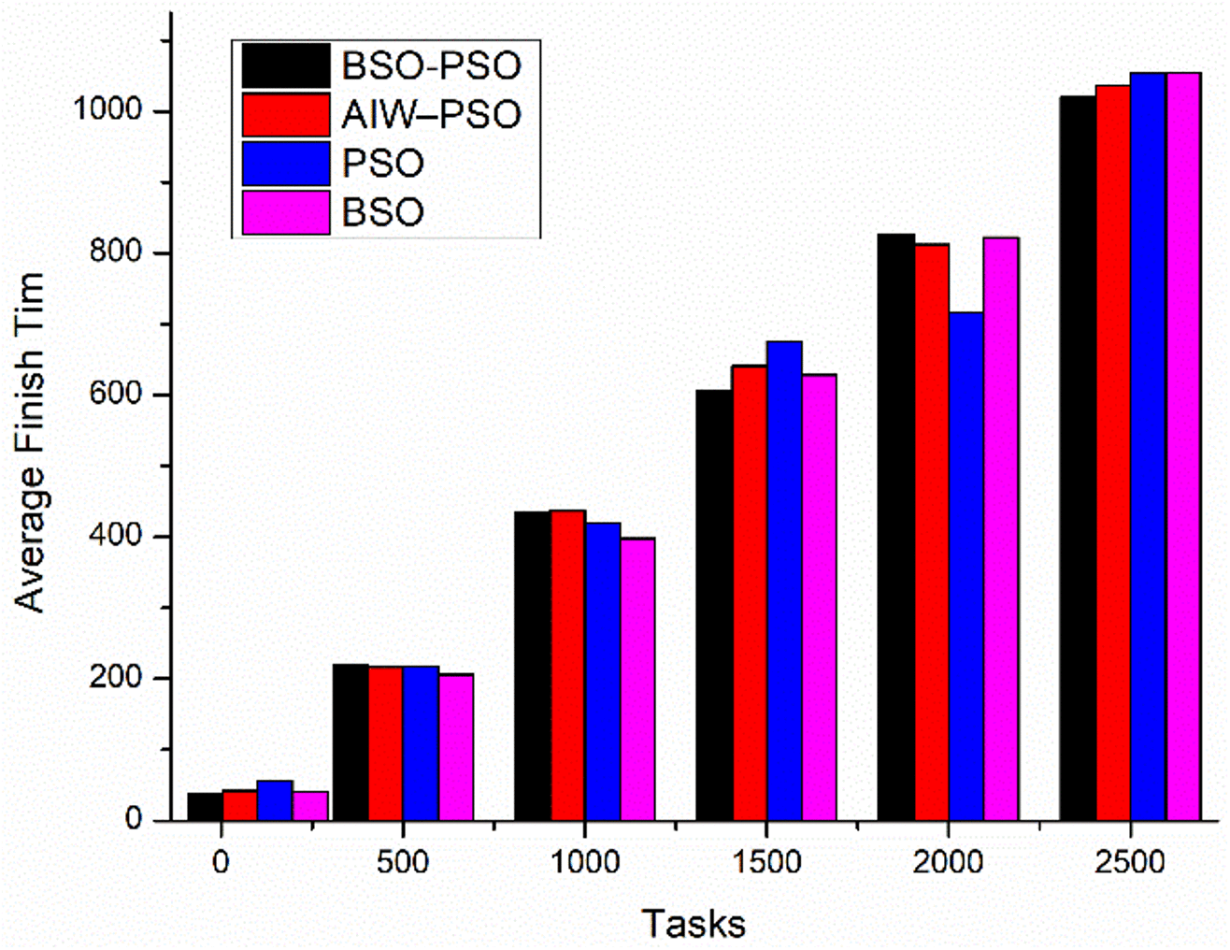
Comparison of Power consumption(kwh).

**Fig 15:**
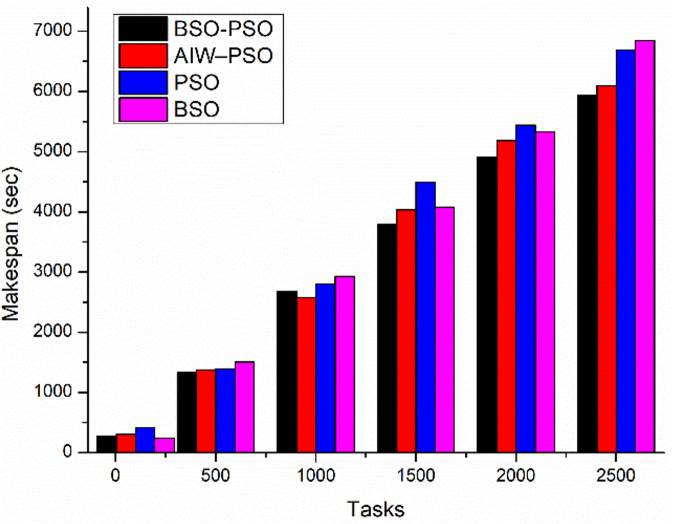
Comparison of Makespan with scaling tasks.

**Fig 16:**
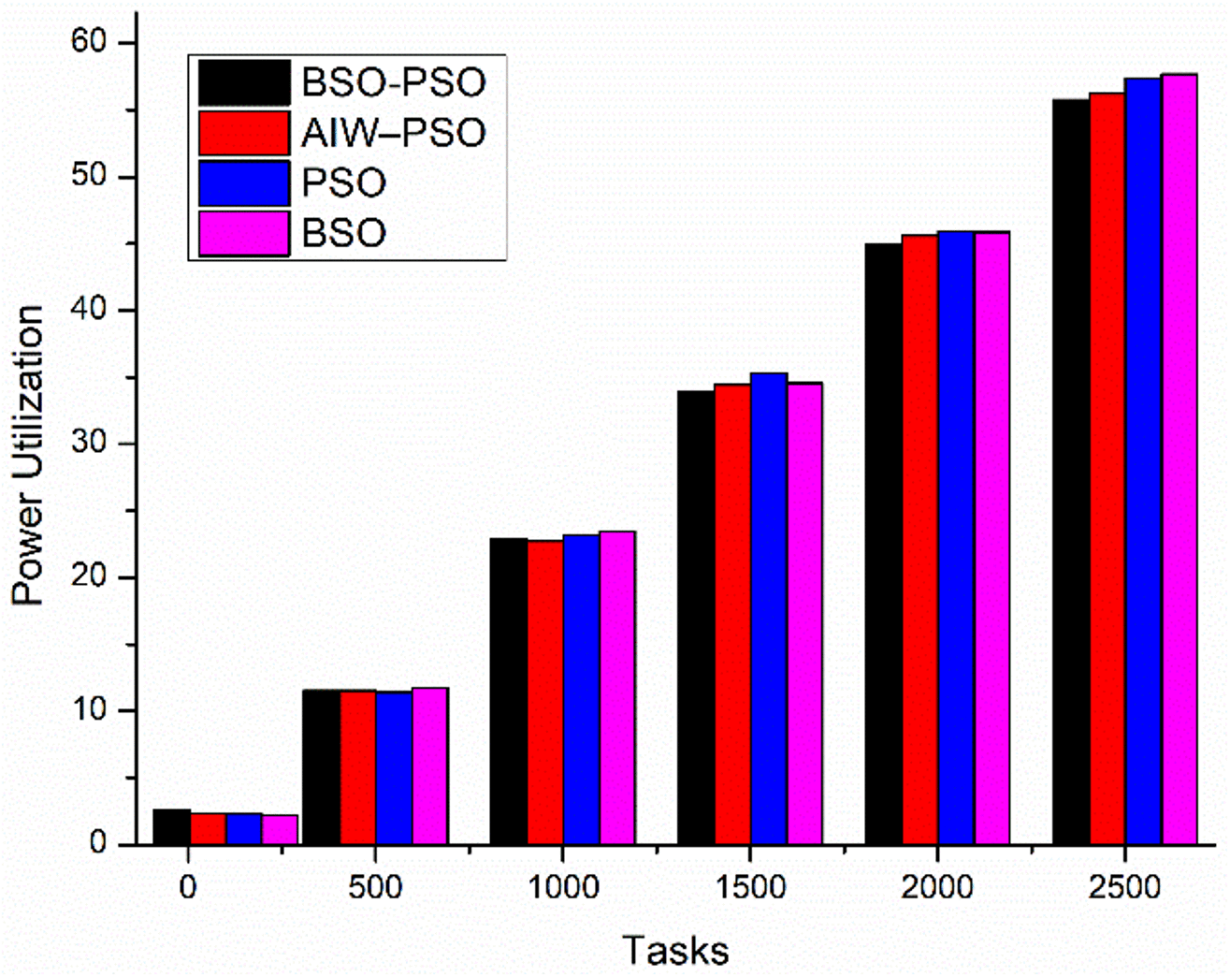
Comparison of average finish time.

**Fig 17:**
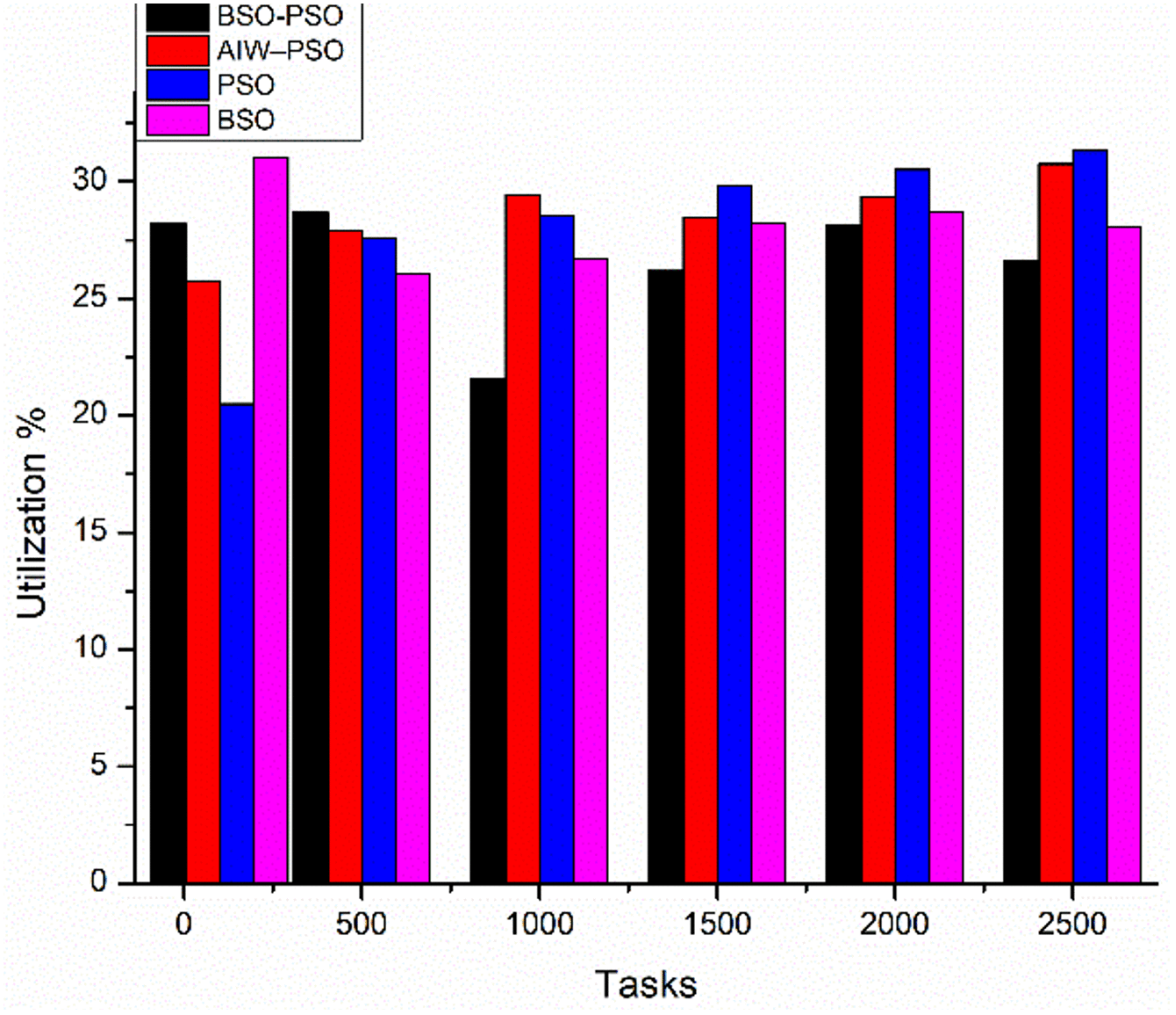
Comparison of utilization with scaling tasks.

The scenario aims to study the performance of the proposed model taking in to considera-tion multi objective fitness function of utilization and makespan all together. Figs 14 and 15 shows the comparative study of average finish time and makespan with changing load. The result shows the improvement in makespan with increasing load and small improvement in average finish time as compared to existing algorithms. On the other hand Figs 16 and 17 shows the performance study of power efficiency and utilization. Where the proposed model out performs the exiting algorithm with reduced power consumption and utilization.

The study and comparative analysis of all 3 scenarios demonstrates that the proposed algorithm fits all type of fitness function and also improves the performance of multiple parameters like energy, utilization, Makespan and finish time all together. The result also shows the proposed algorithm does not sticks in local mina problem which other traditional model suffers from. Also due to this the proposed model is able to find the solution is less time and a global best solution.

Table 6 shows the results of T-Test performed for multi objective function. The result shows Makespan & Power: BSO-PSO wins convincingly against all three — Very Large effect sizes (|d| > 1.4), Finish Time: BSO-PSO beats AIW-PSO and BSO, but PSO has a statistically significant edge (p = 0.0096, Medium effect), Utilization: BSO-PSO is significantly low. Table 7 shows the T-Test result for the utilization fitness function. BSO-PSO dominates across Makespan, Finish Time, and Power with Large to Very Large effects.

**Table 6:** T-test results for multi objective fitness function.

*n = 21 task levels per algorithm | Two-tailed paired t-test | Significance level: α = 0.05*				
Metric	Comparison	t-statistic	p-value	Significant?
**Utilization**	BSO-PSO vs AIW-PSO	−7.8869	0.000000	**Yes**
	BSO-PSO vs PSO	−6.4811	0.000003	**Yes**
	BSO-PSO vs BSO	−7.4721	0.000000	**Yes**
**Makespan**	BSO-PSO vs AIW-PSO	−6.8251	0.000001	**Yes**
	BSO-PSO vs PSO	−7.1317	0.000001	**Yes**
	BSO-PSO vs BSO	−7.0468	0.000001	**Yes**
**Finish Time**	BSO-PSO vs AIW-PSO	−6.6644	0.000002	**Yes**
	BSO-PSO vs PSO	2.8642	0.009590	**Yes**
	BSO-PSO vs BSO	−5.4894	0.000023	**Yes**
**Power**	BSO-PSO vs AIW-PSO	−6.4298	0.000003	**Yes**
	BSO-PSO vs PSO	−6.6905	0.000002	**Yes**
	BSO-PSO vs BSO	−6.8282	0.000001	**Yes**

**Table 7:** T-test results for utilization based fitness function.

Metric	Comparison	t-statistic	p-value	Significant?
**Utilization**	BSO-PSO vs AIW–PSO	−7.5437	0.000000	**Yes**
	BSO-PSO vs PSO	−1.3900	0.179794	No
	BSO-PSO vs BSO	−8.0150	0.000000	**Yes**
**Makespan**	BSO-PSO vs AIW–PSO	−7.6417	0.000000	**Yes**
	BSO-PSO vs PSO	−6.4629	0.000003	**Yes**
	BSO-PSO vs BSO	−6.7533	0.000001	**Yes**
**Finish Time**	BSO-PSO vs AIW–PSO	−6.3074	0.000004	**Yes**
	BSO-PSO vs PSO	−6.1490	0.000005	**Yes**
	BSO-PSO vs BSO	−6.0760	0.000006	**Yes**
**Power**	BSO-PSO vs AIW–PSO	−7.6380	0.000000	**Yes**
	BSO-PSO vs PSO	−6.4655	0.000003	**Yes**
	BSO-PSO vs BSO	−6.7673	0.000001	**Yes**

### 6.4. Discussion

The experimental results show that the proposed Hybrid BCO-PSO algorithm gave measurable and consistent improvements over the standalone metaheuristics algorithm in the case of fog computing load balancing. Across all evaluated workloads (100–10000 tasks), the proposed Hybrid BCO–PSO achieved an average makespan reduction of approximately 18–22% compared to AIW–PSO and 2–4% compared to standard PSO, with the most significant gains observed under high-load conditions. In terms of makespan, the hybrid achieved the lowest or near-lowest values across all workloads, with the most significant gains observed in high-load scenarios of 5000 and 10000 tasks. For example, in the 10000-task case, it reduced makespan by over 35% on compared to the AIW-PSO and showed better results from PSO by 1.61%. These results are achieved by combining BCO’s tumble–swim process, which maintains solution diversity, with PSO’s velocity-based updates that accelerate convergence towards effective task–VM assignments. This allowed the algorithm to adapt effectively to the large search space of high-load cases. Other PSO-based hybrid approaches, such as PSO–Firefly and PSO–Simulated Annealing, have also been reported to improve the fog scheduling performance. Compared to these methods, the proposed BCO–PSO have greater emphasis on exploration through chemotaxis while retaining the controlled PSO updates for fast convergence that is shown in its strong performance under high-load conditions.

Average VM utilisation results show that the hybrid BCO-PSO is having the higher utilisation in most of the scenarios and peaks at 77.93% for 200 tasks, unlike the case experienced by AIW-PSO under heavy workloads (e.g., 47.89% for 10000 tasks). This means that the algorithm spreads tasks more evenly across different types of VMs due to its exploration method. It first considers the capacity of each VM and then adjusts the task distribution to avoid bottlenecks. In real fog computing systems this helps to keep performance steady and give good Quality of Service (QoS).

Execution time analysis shows that in case of small workloads (100 tasks), the hybrid was the fastest method, completing scheduling in 5.18 seconds by converging rapidly to a high-quality solution. In large workloads also it showed good performance with a 351.80second runtime for 10000 tasks compared to 472.67 seconds for AIW-PSO and over 500 seconds for standard PSO. These improvements were achieved by the help from earlier solution stabilisation that reduced the unnecessary computation. In medium workloads (200 and 1000 tasks), PSO was marginally faster due to simpler update rules also this came with slightly higher makespan and lower utilisation. However, the hybrid algorithm also added complexity that may increase the computation overhead when compared to simpler algorithms in medium workloads, and further tuning would be required.

So, the Hybrid BCO-PSO offers a balanced approach that overcomes the stagnation nature of standalone BCO and the premature convergence disadvantage of PSO. The results show that the algorithm performs effectively within fog-inspired, computation-focused scheduling scenarios. It also highlights how combining different metaheuristics can improve performance in several areas.

**Limitations.** This evaluation uses synthetic workloads within a computation-focused fog-inspired scheduling model and assumes that the tasks are independent. Network effects such as latency, bandwidth, and packet loss are not modelled, so the results mainly reflect computation-side load balancing. In real deployments, network and QoS constraints could also change the best scheduling decisions, so the improvements reported here should be interpreted as performance under computation-focused conditions. Although Average VM Utilisation is analysed as a performance metric, it is not included as a competing objective in the optimisation process. As optimisation is performed offline over fixed workloads, the approach is not intended for sub-second real-time task streaming in live fog deployments.

## 7. Conclusion and future work

This study addressed the research question of whether a hybrid metaheuristic can improve load balancing in fog computing by reducing makespan and increasing VM utilisation. The objective was to design, implement, and evaluate a hybrid BCO-PSO algorithm that take advantage of the exploration capability of Bacterial Colony Optimization and the fast convergence nature of Particle Swarm Optimization. Implemented in Python with the custom-developed modules, this algorithm was tested on synthetic task-VM datasets ranging from 100 to 10,000 tasks. The results showed that the hybrid model consistently outperformed the standalone BCO, standard PSO, and AIW–PSO in most scenarios with notable gains under higher workloads. These findings show that the effectiveness of the approach for heterogeneous fog systems, even its current evaluation is limited to the synthetic datasets and does not model network effects.

Future work will focus on improving the adaptability and real-world application. One direction is for adaptive parameter tuning, where chemotaxis step size and PSO velocity weights would adjust dynamically based on the convergence trends. The method can also be extended to multi-objective optimisation and can jointly minimize makespan, Utilization, Task failure count and energy consumption to address the observed performance-energy trade-off. Testing under extreme workload patterns, including high task-size variance and bursty arrivals, would further assess the stability in real-time scenarios. Including fault tolerance into the hybrid update process could also improve in case of failures. Finally, migrating to a simulation framework such as CloudSim Plus would allow the modelling of latency, bandwidth, and QoS parameters, and would provide the realistic, network-aware validation and potential commercial deployment in real-world applications.

## References

[1] Sulimani H, Sulimani R, Ramezani F, Naderpour M, Huo H, Jan T, et al. HybOff: a hybrid offloading approach to improve load balancing in fog environments. J Cloud Comp. 2024;13(1). doi: 10.1186/s13677-024-00663-3

[2] Ogundoyin SO, Kamil IA. Optimal fog node selection based on hybrid particle swarm optimization and firefly algorithm in dynamic fog computing services. Eng Appl Artif Intell. 2023;121:105998. doi: 10.1016/j.engappai.2023.105998

[3] Shaik MB, Subba Reddy K, Chokkanathan K, Asad Ali Biabani S, Shanmugaraja P, Denslin Brabin DR. A hybrid particle swarm optimization and simulated annealing with load balancing mechanism for resource allocation in fog-cloud environments. IEEE Access. 2024;12:172439–50. doi: 10.1109/access.2024.3489960

[4] Niu B, Wang H. Bacterial colony optimization. Discrete Dynam Nat Soc. 2012;2012(1). doi: 10.1155/2012/698057

[5] Tamilarisi K, Gogulkumar M, Velusamy K. Data clustering using bacterial colony optimization with particle swarm optimization. In: 2021 Fourth International Conference on Electrical, Computer and Communication Technologies (ICECCT). IEEE; 2021. pp. 1–5. doi: 10.1109/icecct52121.2021.9616695

[6] Mukherjee M, Shu L, Wang D. Survey of fog computing: fundamental, network applications, and research challenges. IEEE Commun Surv Tutorials. 2018;20(3):1826–57. doi: 10.1109/comst.2018.2814571

[7] Mouradian C, Naboulsi D, Yangui S, Glitho RH, Morrow MJ, Polakos PA. A comprehensive survey on fog computing: state-of-the-art and research challenges. IEEE Commun Surv Tutorials. 2018;20(1):416–64. doi: 10.1109/comst.2017.2771153

[8] Naha RK, Garg S, Georgakopoulos D, Jayaraman PP, Gao L, Xiang Y, et al. Fog computing: survey of trends, architectures, requirements, and research directions. IEEE Access. 2018;6:47980–8009.

[9] Ahmad N, Javaid N, Mehmood M, Hayat M, Ullah A, Khan HA. Fog-Cloud Based Platform for Utilization of Resources Using Load Balancing Technique. International Conference on Network-Based Information Systems (NBiS). Cham: Springer International Publishing. 2018. pp. 554–67. doi: 10.1007/978-3-319-98530-5_48

[10] Abbasi SH, Javaid N, Ashraf MH, Mehmood M, Naeem M, Rehman M. Load Stabilizing in Fog Computing Environment Using Load Balancing Algorithm. International Conference on Broadband and Wireless Computing, Communication and Applications (BWCCA). Cham: Springer International Publishing; 2018. pp. 737–50. doi: 10.1007/978-3-030-02613-4_66

[11] Kaur M, Aron R. A systematic study of load balancing approaches in the fog computing environment. J Supercomput. 2021;77(8):9202–47. doi: 10.1007/s11227-020-03600-8

[12] Singh SP, Kumar R, Sharma A, Nayyar A. Leveraging energy‐efficient load balancing algorithms in fog computing. Concurr Comput. 2020;34(13). doi: 10.1002/cpe.5913

[13] Freitas D, Lopes LG, Morgado-Dias F. Particle swarm optimisation: a historical review up to the current developments. Entropy (Basel). 2020;22(3):362. doi: 10.3390/e22030362 33286136 PMC7516836

[14] Hussein MK, Mousa MH. Efficient task offloading for iot-based applications in fog computing using ant colony optimization. IEEE Access. 2020;8:37191–201. doi: 10.1109/access.2020.2975741

[15] Baburao D, Pavankumar T, Prabhu CSR. Load balancing in the fog nodes using particle swarm optimization-based enhanced dynamic resource allocation method. Appl Nanosci. 2021;13(2):1045–54. doi: 10.1007/s13204-021-01970-w

[16] Reddy PL, Guduri Y. A hybrid bacterial foraging-particle swarm optimization technique for solving optimal reactive power dispatch problem. Int J Appl Power Eng. 2020;9(2):127. doi: 10.11591/ijape.v9.i2.pp127-134

[17] Pradhan A, Bisoy SK. A novel load balancing technique for cloud computing platform based on PSO. J King Saud Univ - Comput Inform Sci. 2022;34(7):3988–95. doi: 10.1016/j.jksuci.2020.10.016

[18] Juneja M, Nagar SK. Particle swarm optimization algorithm and its parameters: A review. In: 2016 International Conference on Control, Computing, Communication and Materials (ICCCCM). IEEE; 2016. 1–5.

[19] Pournazari J, Ullah A, Al-Dubai A, Liu X, Khaledian N. Multi-objective optimisation for energy-centric offloading in fog computing. Computing. 2025;107(11). doi: 10.1007/s00607-025-01578-w

[20] Abdel-Basset M, Mohamed R, Elhoseny M, Bashir AK, Jolfaei A, Kumar N. Energy-Aware Marine Predators Algorithm for Task Scheduling in IoT-Based Fog Computing Applications. IEEE Trans Ind Inf. 2021;17(7):5068–76. doi: 10.1109/tii.2020.3001067

[21] Abdel-Basset M, Moustafa N, Mohamed R, Elkomy OM, Abouhawwash M. Multi-objective task scheduling approach for fog computing. IEEE Access. 2021;9:126988–7009. doi: 10.1109/access.2021.3111130

[22] Wu C, Li W, Wang L, Zomaya AY. Hybrid evolutionary scheduling for energy-efficient fog-enhanced internet of things. IEEE Trans Cloud Comput. 2021;9(2):641–53. doi: 10.1109/tcc.2018.2889482

[23] Ramezani Shahidani F, Ghasemi A, Toroghi Haghighat A, Keshavarzi A. Task scheduling in edge-fog-cloud architecture: a multi-objective load balancing approach using reinforcement learning algorithm. Computing. 2023;105(6):1337–59. doi: 10.1007/s00607-022-01147-5

[24] Revathi J, Eswaramurthy VP, Padmavathi P. Bacterial Colony Optimization for Data Clustering. In: 2019 IEEE International Conference on Electrical, Computer and Communication Technologies (ICECCT). IEEE; 2019. pp. 1–4. doi: 10.1109/icecct.2019.8869366

[25] Saranya M, Pabitha P. Hybrid Multi-objective Harris-Hawks and Moth-Flame Optimization Algorithm for Efficient Task Offloading strategy in IoT-Based Fog Computing Applications. In: 2024 International Conference on Knowledge Engineering and Communication Systems (ICKECS). IEEE; 2024. pp. 1–6. doi: 10.1109/ickecs61492.2024.10616445

[26] Sabat NR, Sahoo RR, Pradhan MR, Acharya B. Hybrid technique for optimal task scheduling in cloud computing environments. Telecommun Comput Electron Control. 2024;22(2):380–92.

[27] Janakiraman S, Priya MD. Improved Artificial Bee Colony Using Monarchy Butterfly Optimization Algorithm for Load Balancing (IABC-MBOA-LB) in Cloud Environments. J Netw Syst Manage. 2021;29(4). doi: 10.1007/s10922-021-09602-y

[28] Qiao J, Wang G, Yang Z, Luo X, Chen J, Li K, et al. A hybrid particle swarm optimization algorithm for solving engineering problem. Sci Rep. 2024;14(1):8357. doi: 10.1038/s41598-024-59034-2 38594511 PMC11375002

[29] Chandak A, Ray NK. A Review of Load Balancing in Fog Computing. In: 2019 International Conference on Information Technology (ICIT). IEEE; 2019. pp. 460–5. doi: 10.1109/icit48102.2019.00087

[30] Nickabadi A, Ebadzadeh MM, Safabakhsh R. A novel particle swarm optimization algorithm with adaptive inertia weight. Appl Soft Comput. 2011;11(4):3658–70. doi: 10.1016/j.asoc.2011.01.037

[31] Van Thieu N, Mirjalili S. MEALPY: An open-source library for latest meta-heuristic algorithms in Python. J Syst Arch. 2023;139:102871. doi: 10.1016/j.sysarc.2023.102871

